# Expression of Aldo-Keto Reductase Family 1 Member B10 in the Early Stages of Human Hepatocarcinogenesis

**DOI:** 10.3390/ijms15046556

**Published:** 2014-04-17

**Authors:** Hironori Tsuzura, Takuya Genda, Shunsuke Sato, Ayato Murata, Yoshio Kanemitsu, Yutaka Narita, Sachiko Ishikawa, Tetsu Kikuchi, Masashi Mori, Katsuharu Hirano, Katsuyori Iijima, Ryo Wada, Takafumi Ichida

**Affiliations:** 1 Department of Gastroenterology and Hepatology, Juntendo University Shizuoka Hospital, 1129 Nagaoka, Izunokuni-shi, Shizuoka 410-2295, Japan; E-Mails: htudura@juntendo.ac.jp (H.T.); syusato@juntendo.ac.jp (S.S.); perseverance3@hotmail.co.jp (A.M.); yoshio-k.s52@live.jp (Y.K.); ynarita@juntendo.ac.jp (Y.N.); s-ogawa@juntendo.ac.jp (S.I.); klinsmann1011@yahoo.co.jp (T.K.); ten2617@gmail.com (M.M.); khirano-tym@umin.ac.jp (K.H.); katsu0716@shore.ne.jp (K.I.); takafumi@air.ocn.ne.jp (T.I.); 2 Department of Pathology, Juntendo University Shizuoka Hospital, 1129 Nagaoka, Izunokuni-shi, Shizuoka 410-2295, Japan; E-Mail: mdksmrwjunten522@ybb.ne.jp

**Keywords:** AKR1B10, HSP70, glypican-3, hepatocellular carcinoma, chronic hepatitis, cirrhosis

## Abstract

Aldo-keto reductase family 1, member B10 (AKR1B10), a cancer-related oxidoreductase, is expressed in well-differentiated hepatocellular carcinomas (HCCs). However, AKR1B10 levels are minimal in normal liver tissues (NLs), similar to the 70-kilodalton heat shock protein (HSP70) and glypican-3. Moreover, the role of AKR1B10 in chronic hepatitis or cirrhosis, which are considered preneoplastic conditions for HCC, has not been fully elucidated. The aim of this study was to evaluate the expression of AKR1B10, HSP70, and glypican-3 in 61 HCC tissue samples compared to corresponding non-tumorous liver tissues (NTs), comprising 42 chronic hepatitis and 19 cirrhosis cases to clarify the significance of molecular changes at the preneoplastic stages of HCC. Immunohistochemical analysis demonstrated that the median expression levels of AKR1B10 were higher in HCCs than in NTs (*p* < 0.001) and higher in NTs than NLs (*p* < 0.001) with 54.8%, 2.1%, and 0.3% expression in HCCs, NTs, and NLs, respectively. HSP70 and glypican-3 were expressed in HCCs, but minimally in NTs and NLs with no significant difference between expression in NTs and NLs. Furthermore, a multivariate analysis identified an association between hepatic steatosis and AKR1B10 expression in NTs (*p* = 0.020). Of the three protein expressed in well-differentiated HCCs, only AKR1B10 was upregulated in preneoplastic conditions, and a steatosis-related factor might influence its expression.

## 1. Introduction

Hepatocellular carcinoma (HCC) is the fifth most common cancer and the third most common cause of cancer-related death worldwide [1]. HCC is characterized by a multistep morphological developmental process, progressing from early, well-differentiated HCC to moderately or poorly differentiated, advanced HCC [2]. Until now, several molecular alterations have been found to be associated with morphological developments in HCC. The 70-kilodalton heat shock protein (HSP70) and glypican-3 (GPC3) are upregulated even in early, well-differentiated HCC [3,4], while accumulations of mutated beta-catenin and p53 are mainly observed in moderately to poorly differentiated HCC [5,6]. On the other hand, the majority of HCCs arise in chronically diseased livers, including livers with chronic hepatitis and cirrhosis resulting from hepatitis B or C infection or exposure to other carcinogenic factors. These chronic liver diseases are widely considered to be preneoplastic conditions for HCC, potentially leading to molecular alterations that predispose hepatocytes to malignant transformation. However, the molecular alterations underlying preneoplastic conditions that predispose to HCC remain poorly understood.

Aldo-keto reductase family 1 member B10 (AKR1B10) is a member of the AKR superfamily, which are NAD(P)H-dependent oxidoreductases that catalyze the reduction of carbonyl compounds and various physiological and xenobiotic substrates [7,8]. AKR1B10 was originally isolated as a gene with increased expression in human HCC [9,10], including well-differentiated HCC, but is minimal in normal liver tissue [11,12]. This is similar to findings regarding the expression of HSP70 and GPC3. Recently, we analyzed the expression profiles of approximately 41,000 genes in patients with chronic hepatitis C and found that AKR1B10 was upregulated in the livers of chronic hepatitis C patients at high risk of HCC [13]. These observations suggest that an alteration of AKR1B10 expression occurs even in preneoplastic conditions that predispose to HCC. To further study the significance of AKR1B10 alterations in the early stages of hepatocarcinogenesis, we evaluated AKR1B10 expression in 61 HCCs and corresponding non-tumorous liver tissues (NTs), which comprised 42 chronic hepatitis and 19 cirrhosis cases, and compared AKR1B10 expression with the expression of the other two molecules known to be upregulated in well-differentiated HCC—HSP70 and GPC3—both of which are widely used as immunohistochemical molecular markers of early HCC [14]. In addition, because a strong association has been reported between smoking and AKR1B10 expression in malignant and non-malignant lung airway epithelium [15–17] and several studies have identified smoking as a risk factor for HCC development [18,19], we further evaluated the correlation of AKR1B10 expression with NT-associated factors, including patients’ smoking history.

## 2. Results

### 2.1. Clinicopathological Patient Features

Table 1 summarizes demographic, biochemical, and pathological data for all 61 patients enrolled in this study. Serological markers of hepatitis virus were distributed as follows: positive for HBsAg, *n* = 13 (21%); positive for anti-HCV (hepatitis C virus), *n* = 33 (54%); and negative for HBsAg and anti-HCV, *n* = 15 (25%). The median HCC size was 48 mm (range: 12–170 mm), and 19 HCCs were classified as well differentiated, 38 were moderately differentiated, and 4 were poorly differentiated. The distribution of the degree of hepatic fibrosis in NTs was as follows: no fibrosis or periportal fibrous expansion (F0–1), *n* = 18; portal fibrous widening with bridging fibrosis (F2), *n* = 14; bridging fibrosis with lobular distortion (F3), *n* = 10; and liver cirrhosis (F4), *n* = 19. Steatosis in NTs ranged between 0.0% and 23.3% (median 1.3%).

### 2.2. Immunohistochemical Analyses

**Figure 1.**
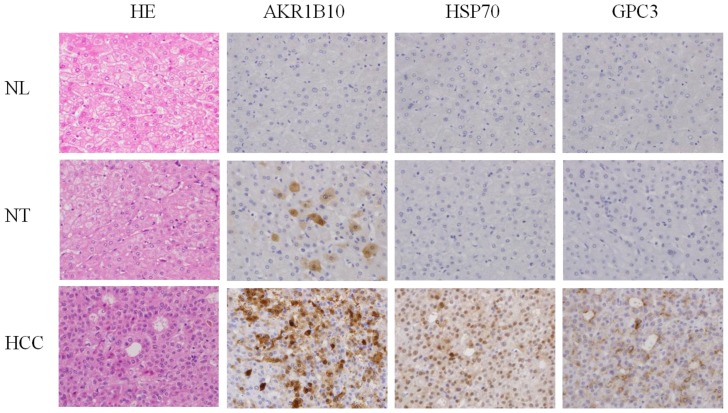
Representative immunostaining of aldo-keto reductase family 1 member B10 (AKR1B10), 70-kilodalton heat shock protein (HSP70), and glypican-3 (GPC3). HCC, hepatocellular carcinoma; HE, hematoxylin-eosin; NL, control normal liver tissue; NT, non-tumorous liver tissue. Original magnification ×100.

Figure 1 shows representative immnohistochemical staining of AKR1B10, HSP70, and GPC3 in liver tissues. No AKR1B10 immunoreactivity was observed in any of the 8 NLs, but there was detectable nucleocytoplasmic AKR1B10 immunoreactivity in single, scattered hepatocytes or some clustered hepatocytes in 44 of 61 NTs. More widespread nucleocytoplasmic immnoreactivity of AKR1B10 was seen in 55 of the 61 HCCs. In addition, HSP70 immunoreactivity was observed in 50 of the 61 HCCs and GPC3 immnoreactivity in 42 of the 61 HCCs. However, HSP70 and GPC3 immunoreactivity was minimal in both NTs and NLs.

**Figure 2.**
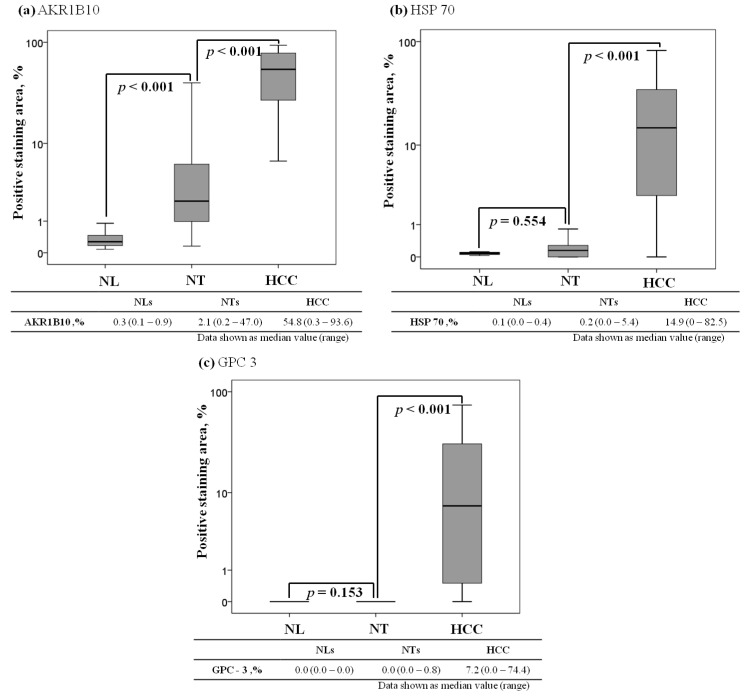
Comparison of AKR1B10, HSP70, and GPC3 expression levels among control normal liver tissues (NLs), non-tumorous liver tissues (NTs), and hepatocellular carcinomas (HCCs). The box encompasses the 25th through 75th percentiles, and the horizontal line through the middle of the box indicates the fiftieth percentile (median). The 10th and 90th percentiles are shown as whisker caps. (**a**) AKR1B10 expression; (**b**) HSP70 expression; and (**c**) GPC3 expression.

Quantification of the immunoreactivity by image analyses showed areas staining positive for AKR1B10 ranging from 0.3% to 93.6% (median 54.8%) in HCCs, 0.2% to 47.0% (median 2.1%) in NTs, and 0.1% to 0.9% (median 0.3%) in NLs. The AKR1B10 expression level was significantly higher in HCCs than in NTs (*p* < 0.001) and higher in NTs than in NLs (*p* < 0.001, Figure 2A). The HSP70 expression level ranged between 0.0% and 82.5% (median 14.9%) in HCCs, 0.0% and 5.4% (median 0.2%) in NTs, and 0.0% and 0.4% (median 0.1%) in NLs. The HSP70 expression level was significantly higher in HCCs than in NTs (*p* < 0.001), but did not differ significantly between NTs and NLs (*p* = 0.80, Figure 2B). The GPC3 expression level ranged between 0.0% and 74.4% (median 7.2%) in HCCs, 0.0% and 0.8% (median 0.0%) in NTs, and 0.0% and 0.0% (median 0.0%) in NLs. The GPC3 expression level was significantly higher in HCCs than in NTs (*p* < 0.001), but did not differ significantly between NTs and NLs (*p* = 0.80, Figure 2C).

### 2.3. Factors Associated with Aldo-Keto Reductase Family 1 Member B10 (AKR1B10) Expression in Non-Tumorous Liver Tissues (NTs)

**Figure 3.**
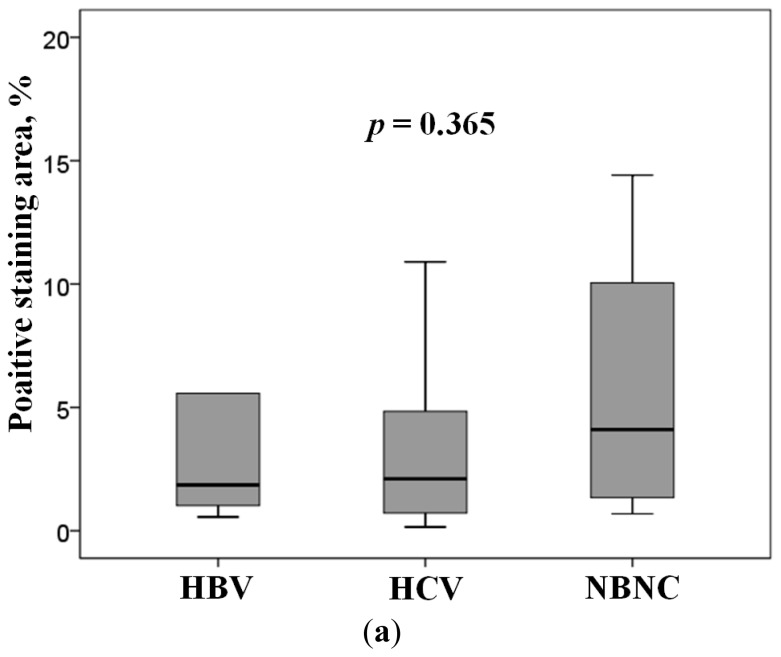

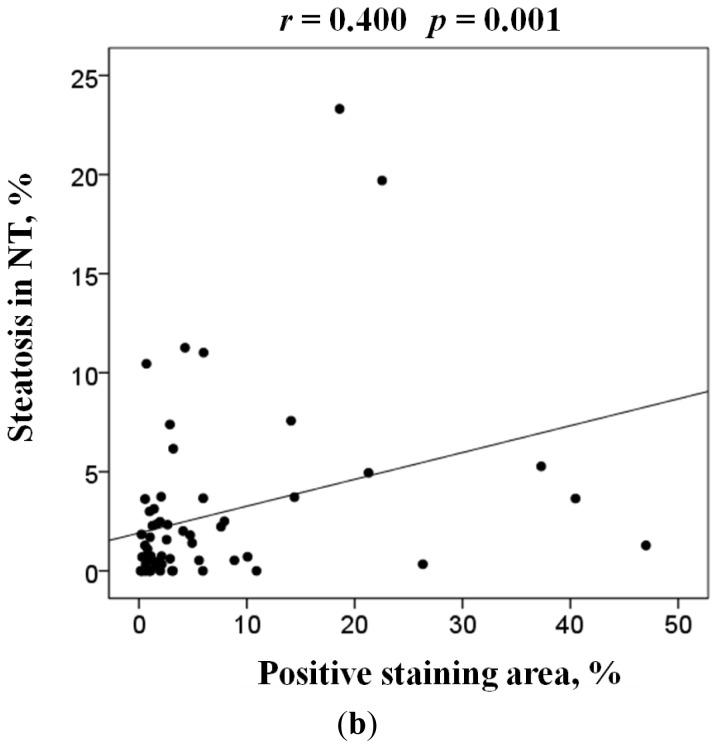
(**a**) ARK1B10 expression level in non-tumorous liver tissue. HBV, positive for HBsAg; HCV, positive for anti-HCV; NBNC, negative for HBsAg and anti-HCV. The box encompasses the twenty-fifth through seventy-fifth percentiles, and the horizontal line through the middle of the box indicates the fiftieth percentile (median). The tenth and ninetieth percentiles are shown as whisker caps; and (**b**) Regression analysis of the relationship between AKR1B10 expression and hepatic steatosis in non-tumorous liver tissues.

**Figure 4.**
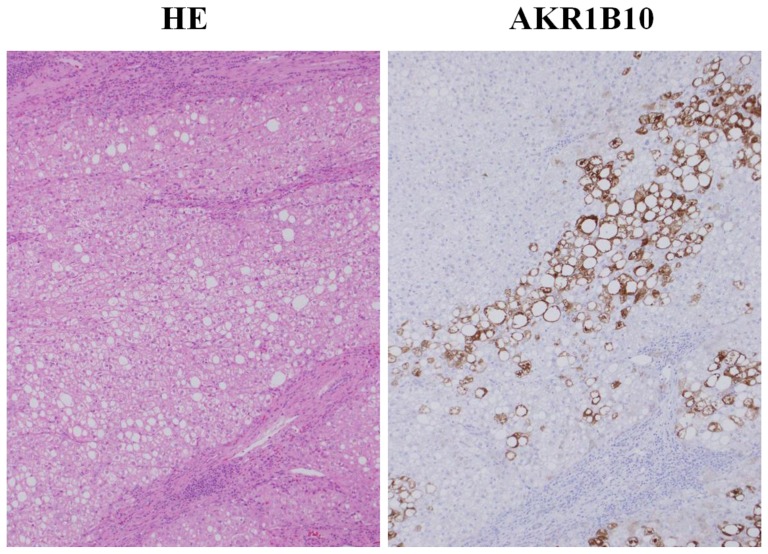
Representative immunostaining of AKR1B10 in hepatocytes containing fatty change. AKR1B10, aldo-keto reductase family 1 member B10; HE, hematoxylin-eosin. Original magnification ×100.

Of the 3 molecules upregulated in well-differentiated HCC, only AKR1B10 expression increased in NTs. Therefore, we next evaluated the association between AKR1B10 expression and various clinicopathological parameters in NTs (Table 2). In the univariate analysis, patients’ clinical and biochemical factors, viral markers, fibrosis stage of NTs, and grade of HCC were not associated with AKR1B10 expression in NTs, and hepatic steatosis was the only parameter significantly associated with AKR1B10 expression. The multivariate analysis confirmed this significant association between AKR1B10 expression and hepatic steatosis (*p* = 0.020). Figure 3A shows a comparison of AKR1B10 expression levels in NTs among HBsAg positive, anti-HCV positive, and HBsAg and anti-HCV negative patients. Median AKR1B10 expression levels in these 3 patient groups were 1.9%, 2.1%, and 4.1%, respectively. AKR1B10 expression tended to be higher in HBsAg and anti-HCV negative patients, but the difference was not statistically significant. Figure 3B shows the regression analysis of AKR1B10 expression *versus* hepatic steatosis in NTs, with a significant positive correlation between the 2 (*r* = 0.40, *p* = 0.001). Our immnohistochemical examination also found a tendency towards more prominent AKR1B10 immunoreactivity in hepatocytes showing fatty change than in hepatocytes with no fatty change (Figure 4).

## 3. Discussion

In the present study, we demonstrated that AKR1B10 expression levels were significantly higher in livers with chronic hepatitis or cirrhosis, which are preneoplastic conditions underlying HCC, than in normal livers, and that AKR1B10 expression was still higher in HCCs. Because the AKR superfamily consists of more than 100 members, and several AKR members, such as AKR1B15, show high amino acid sequence identity with AKR1B10, cross-reactivity of the anti-AKR1B10 antibody used in this study may become an issue in immunohistochemical analysis. However, both our previous study and another study performed quantitative reverse transcription polymerase chain reaction for AKR1B10, and both reported results consistent with AKR1B10 immunohistochemistry [13,20]. HSP70 and GPC3 also showed prominent immunoreactivities in HCC, but minimal immunoreactivities in NTs and NLs, consistent with previous reports [3,4]. These results indicate that HSP70 and GPC3, but not AKR1B10, are useful markers for distinguishing HCC from chronic hepatitis or cirrhosis. However, the stepwise upregulation of AKR1B10 from chronic hepatitis or cirrhosis to HCC might indicate its potential role in the early stage of hepatocarcinogenesis.

Many studies have demonstrated AKR1B10 upregulation in several types of cancer, including several recent reports on HCC [11,20–22]. AKR1B10 was shown to have a high catalytic efficiency for the reduction of all *trans*-, 9-*cis*-, and 13-*cis*-retinals to their corresponding retinols *in vitro* and *in vivo* [23,24], and the conversion of retinals to retinols via AKR1B10 can deprive retinoic acid receptors of their ligands, and can presumably inhibit the retinoic acid signaling pathway [25,26]. Retinoic acid is thought to be essential for the maintenance of normal epithelial differentiation. Retinoic acid depletion causes cell proliferation and loss of differentiation, thereby inducing neoplastic phenotypes in normal epithelium [27–29]. On the other hand, retinoic acid exposure inhibits proliferation of normal and transformed cells *in vitro* [30,31], and dietary retinoic acid reduced the development of premalignant and malignant lesions in a chemically induced mouse carcinogenesis model [32]. AKR1B10 was shown to be downregulated using small, interfering, RNA-inhibited cancer cell proliferation both *in vitro* and *in vivo* [22,33]. Furthermore, oral administration of acyclic retinoids was reported to prevent human HCC [34]. These observations suggest the involvement of AKR1B10 in cancer cell dedifferentiation and proliferation via inhibition of retinoic acid signals. In addition to its hypothetical role in retinoid metabolism, AKR1B10 has been reported to perform other potential functions such as detoxification of toxic aldehydes, fatty acid synthesis, and resistance to carbonyl-containing drugs [15]. These functions may also be involved in the molecular mechanisms underlying carcinogenesis. Consistent with our findings, several reports demonstrated AKR1B10 upregulation in some preneoplastic conditions such as squamous metaplasia and Barrett’s esophagus [15,16,35].

Although several studies have reported that smoking affects AKR1B10 expression in malignant and non-malignant lung airway epithelium [15–17], no association between AKR1B10 expression in the liver and smoking was demonstrated in this study. Interestingly, the present study showed a significant association between AKR1B10 expression in NTs and hepatic steatosis: a cytological change marked by clear vacuoles because of fat accumulation in hepatocytes. Recently, we conducted a study demonstrating that AKR1B10 upregulation was a risk factor for HCC development in chronic hepatitis C patients [13]. Hepatic steatosis *per se* has been considered a risk factor for HCC development. Presence of hepatic steatosis is associated with increased frequency of HCC in patients with HCV-related cirrhosis [36]. Alcoholic and non-alcoholic steatohepatitis is recognized as an important liver disease preceding cirrhosis and HCC [37,38]. Taken together, all of these findings suggest that AKR1B10 might play a role in the molecular basis of steatosis-related hepatocarcinogenesis. Indeed, Starmann *et al.* reported that AKR1B10 expression was upregulated during the progression of simple steatosis to steatohepatitis with increased risk of HCC [39]. The present study showed that AKR1B10 expression in NTs was not significantly different according to hepatitis B or C viral infection status. Interestingly, of the 15 NT liver samples in this study that were negative for hepatitis B surface antigen (HBsAg) and anti-hepatitis C virus antibody (anti-HCV), 9 showed histological features of steatohepatitis. In addition, AKR1B10 expression tended to be higher in HBsAg- and anti-HCV-negative NT samples, compared to HBsAg- or anti-HCV-positive NT samples, although the difference was not statistically significant. These results partially confirm the results reported by Starmann *et al.* Because hepatic steatosis is generally associated with metabolic disorders such as obesity and type 2 diabetes, some metabolic disorders might affect AKR1B10 expression in preneoplastic conditions [40].

## 4. Patients and Methods

### 4.1. Tissue Samples

We obtained paired samples of primary HCCs and their corresponding NTs from 61 patients who underwent hepatic resection at Juntendo University Shizuoka Hospital, Izunokni, Japan between 2004 and 2012. None of the patients were previously treated. The following laboratory parameters were measured using commercially available assays immediately prior to hepatic resection: HBsAg and anti-HCV levels (LUMIPULSE Presto^®^, FUJIREBIO Inc., Tokyo, Japan); blood cell count (COULTER LH780, Beckman coulter, Inc., Brea, CA, USA); prothrombin time (Thromborel S^®^, SYSMEX Co., Kobe, Japan); and serum albumin, alanine aminotransferase (ALT), total bilirubin (AQUAAUTO, KAINOS Laboratories, Inc., Tokyo, Japan), alpha-fetoprotein (ARCHITECT^®^, ABBOT JAPAN Co., Tokyo, Japan), and des-gamma-carboxy prothrombin (BML, Inc., Tokyo, Japan) levels. HCC histological grades were determined according to the World Health Organization criteria [41]. In instances where different tumor grades were found within the same nodule, the predominant histological grade was used. Histological evaluation of NTs was based on the METAVIR criteria, as reported previously [42]. Steatosis in NTs was quantitatively assessed by computer-assisted morphometric image analysis as previously described [43]. Control normal liver tissues (NLs) showing no unusual histological features were also obtained from surgically resected materials obtained from 8 patients with liver metastasis of colorectal cancer.

This study was approved by the Ethical Committee of Juntendo University Shizuoka Hospital in accordance with the Helsinki Declaration, and written informed consent was obtained from all patients.

### 4.2. Immunohistochemistry

Immunohistochemical analyses for AKR1B10, HSP70, and GPC3 were performed on formalin-fixed, paraffin-embedded tissue sections using an immunoperoxidase method. In brief, deparaffinized and rehydrated sections were processed by heat-induced antigen retrieval in 0.1 M citrate buffer at pH 6.0. After blocking endogenous peroxidase activity with 0.3% H_2_O_2_ in methanol solution, the sections were treated with 2% normal swine serum and incubated with primary antibody overnight at room temperature, followed by incubation with biotinylated secondary antibody (Ventana iVIEW DAB Universal Kit; Ventana Medical Systems Inc., Tucson, AZ, USA). Staining was visualized using 3,3′-diaminobenzidine tetrahydrochloride and hematoxylin counterstain. Negative controls were prepared by replacing the primary antibody with mouse immunoglobin (Sigma–Aldrich Biochemicals, St. Louis, MO, USA). Immunostaining was quantitatively assessed as the mean percentage of the positive staining areas in 2 independent fields at 100× magnification using Lumina Vision 2.4 Bio-imaging software (Mitani Corporation, Tokyo, Japan). The following antibodies were used in this study: mouse anti-human AKR1B10 antibody (1:100 dilution; Ab 57547; Abcam, Cambridge, UK), anti-human HSP70 antibody (1:100 dilution; SC-24; Santa Cruz Biotechnology Inc., Santa Cruz, CA, USA), and anti-human GPC3 antibody (1:100 dilution; 1G12, Biomosaics, Burlington, VT, USA).

### 4.3. Statistical Analysis

All statistical analyses were performed using IBM SPSS 13.0 software (IBM SPSS, Chicago, IL, USA). Continuous variables were summarized as median (range), and Mann-Whitney *U*-tests or Kruskal-Wallis tests were used when appropriate. Univariate and multivariate regression analyses were used to examine the relationship of AKR1B10 expression in NT with demographic, histological, and biochemical variables. *p* < 0.05 was considered statistically significant.

## 5. Conclusions

We found that AKR1B10 expression is upregulated in chronic hepatitis or cirrhosis, preneoplastic conditions that predispose to HCC, in association with hepatic steatosis. Our findings could provide insight into the molecular mechanism of the very early stages of human hepatocarcinogenesis and a novel therapeutic target for the prevention of HCC.

## Figures and Tables

**Table 1.** Clinicopathological characteristics of patients included in this study.

Characteristics	Units	*N* = 61
Age	(years) *	67 (38–83)
Sex	(male/female)	47/14
Habitual drinker	(yes/no)	27/34
Smoking	(0/<40/≥40 pack-years)	38/11/12
Hepatitis virus	(B/C/NBNC)	13/33/15
Albumin	(g/mL) *	3.8 (3.0–4.9)
ALT	(IU/L) *	44 (8–224)
Platelet count	(10^4^/μL) *	14.9 (6.0–45.6)
Total bilirubin	(mg/dL) *	0.7 (0.3–1.7)
Prothrombin time	(%) *	91 (68–136)
AFP	(ng/mL) *	13 (2–60,514)
DCP	(mAU/mL) *	184 (0–129,000)
Tumor	size (mm) *	48 (12–170)
Grade of HCC	(W/M/P)	19/38/4
Fibrosis stage of NT	(F0–1/F2/F3/F4)	18/14/10/19
Steatosis in NT	(%) *	1.3 (0.0–23.3)

* Date shown as median value (range).

Abbreviations: AFP, alpha-fetoprotein; ALT, alanine aminotransferase; B, positive for HBsAg; C, positive for anti-HCV; DCP, des-gamma-carboxy prothrombin; HCC, hepatocellular carcinoma; M, moderately differentiated; NBNC, negative for HBsAg and anti-HCV; NT, non-tumorous liver tissue; P, poorly differentiated; W, well differentiated.

**Table 2.** Univariate and multivariate analysis of factors associated with AKR1B10 expression in non-tumorous liver tissues.

Variables	Univariate		Multivariate	
	
	Coefficient	*p* Value	Coefficient	*p* Value
Age	−0.109	0.403		
Male gender	0.033	0.802		
Habitual drinker	0.069	0.598		
Smoking	0.003	0.980		
Hepatitis virus	−0.111	0.395		
Albumin	−0.160	0.218		
ALT	0.133	0.305		
Platelet count	−0.173	0.182		
Total bilirubin	−0.068	0.604		
Prothrombin time	−0.105	0.427		
AFP	−0.107	0.415		
DCP	−0.100	0.466		
Grade of HCC	−0.032	0.805		
Fibrosis stage of NT	0.130	0.318		
Steatosis in NT	0.306	0.004	0.317	0.021

Stepwise linear regression analysis was used in the univariate and multivariate analysis. Abbreviations: AFP, alpha-fetoprotein; ALT, alanine aminotransferase; DCP, des-gamma-carboxy prothrombin; HCC, hepatocellular carcinoma; NT, non-tumorous liver tissue.
